# Supplementary material for: In-Silico Identification of Novel Pharmacological Synergisms: The Trabectedin Case
Source: Int J Mol Sci. 2024 Feb 8;25(4):2059. doi: 10.3390/ijms25042059 (PMC10888651; doi:10.3390/ijms25042059)
Supplement: Supplementary file 1 [file ijms-25-02059-s001.zip › Figure_S1.pdf]

Figure S1
Heatmaps showing the tau score of each pair of dataset-compounds for each condition associated with the dataset. Rows represent the first 10 most correlated compounds, as reported on the right. Columns report each query signature related to the dataset, represented in color in the first colored line and referred to in the legend below. The second color line represents cell lines of the Connectivity Map database, as referred to in the legend below.

A

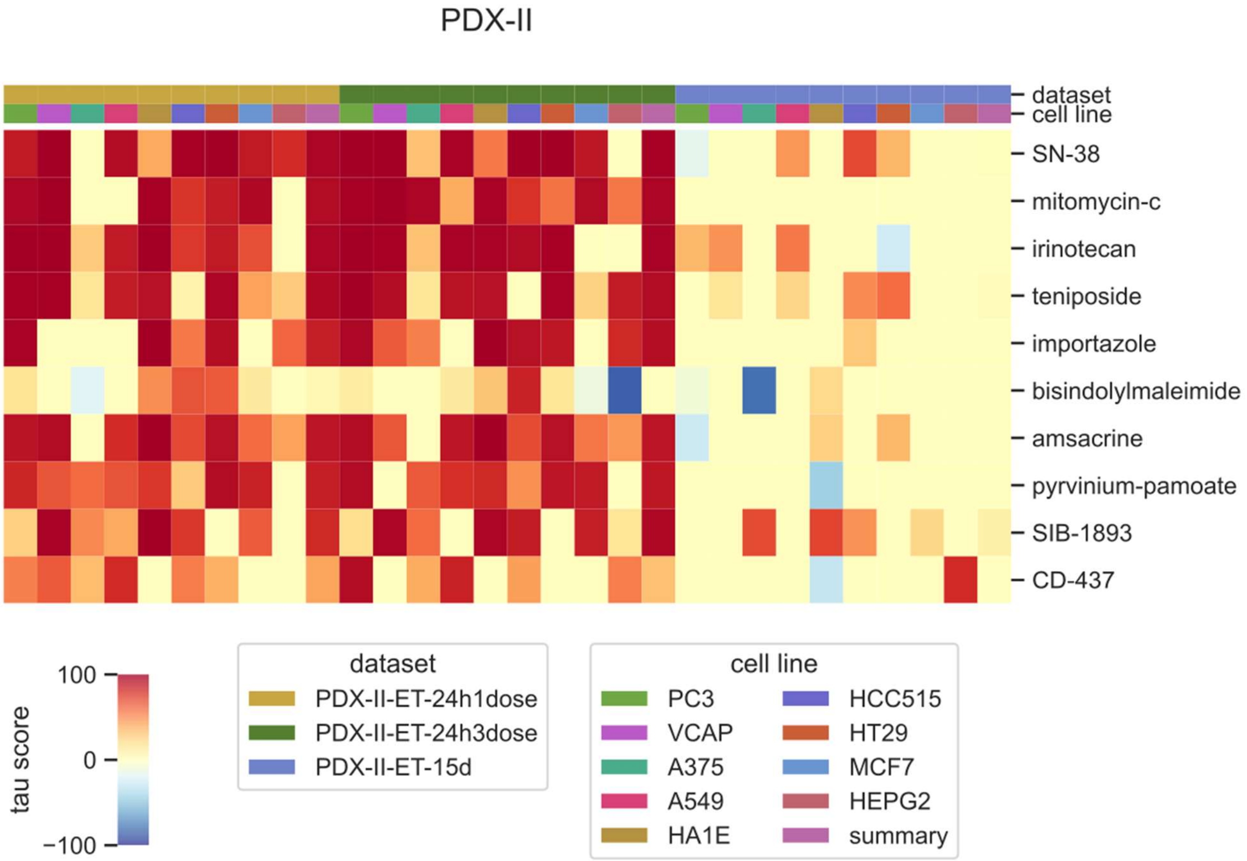

B

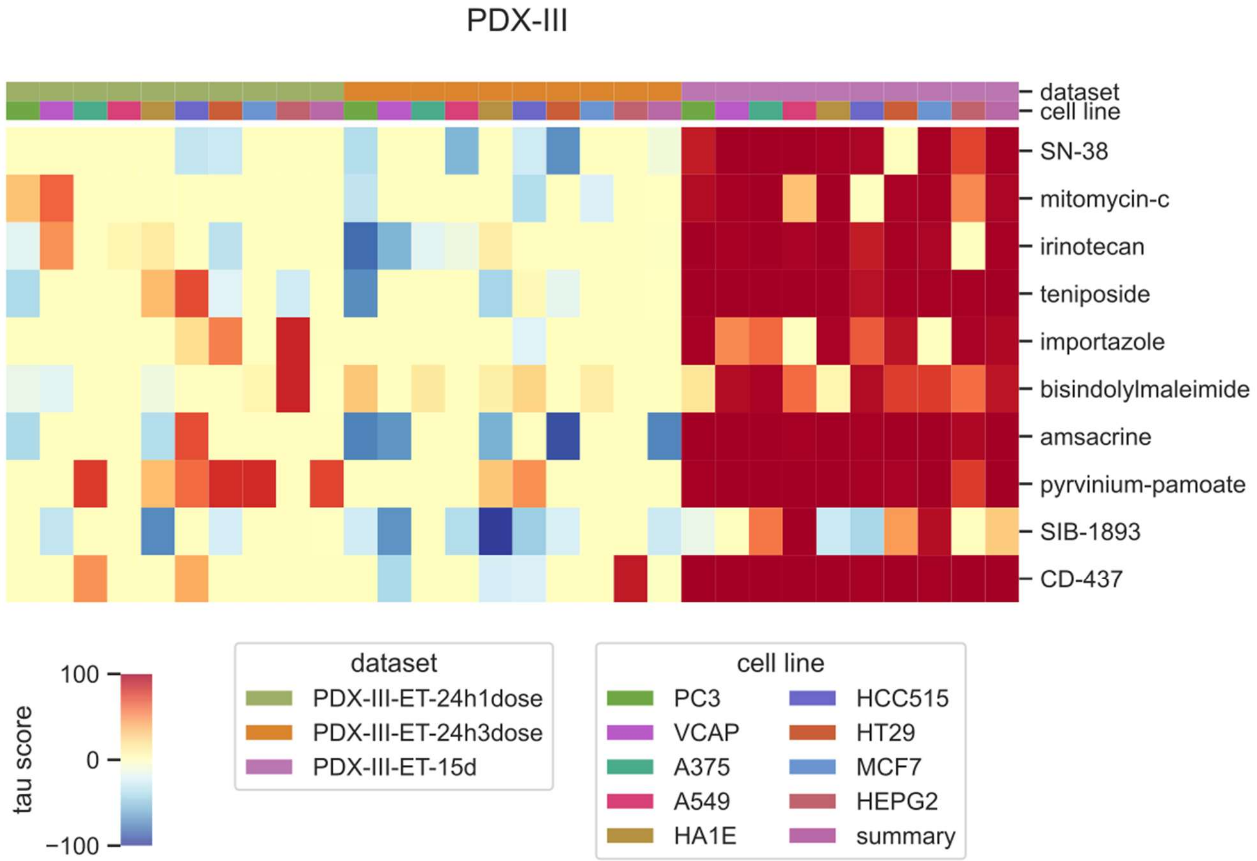
PDX-III
dataset
cell line
SN-38
mitomycin-c
irinotecan
teniposide
importazole
bisindolylmaleimide
amsacrine
pyrvinium-pamoate
SIB-1893
CD-437
tau score
100
0
−100
dataset
PDX-III-ET-24h1dose
PDX-III-ET-24h3dose
PDX-III-ET-15d
cell line
PC3
VCAP
A375
A549
HA1E
HCC515
HT29
MCF7
HEPG2
summary

C

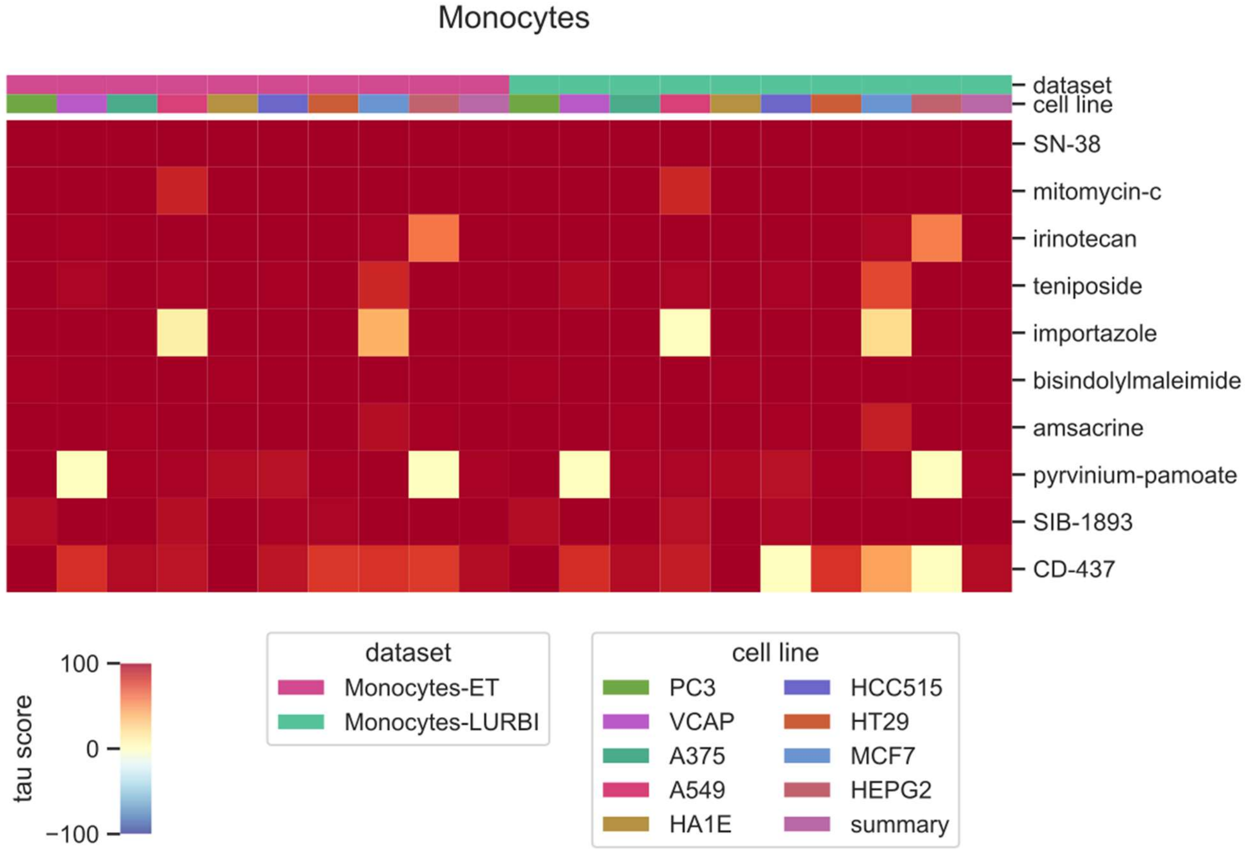
Monocytes
dataset
cell line
SN-38
mitomycin-c
irinotecan
teniposide
importazole
bisindolylmaleimide
amsacrine
pyrvinium-pamoate
SIB-1893
CD-437
tau score
100
0
−100
dataset
Monocytes-ET
Monocytes-LURBI
cell line
PC3
VCAP
A375
A549
HA1E
HCC515
HT29
MCF7
HEPG2
summary

D

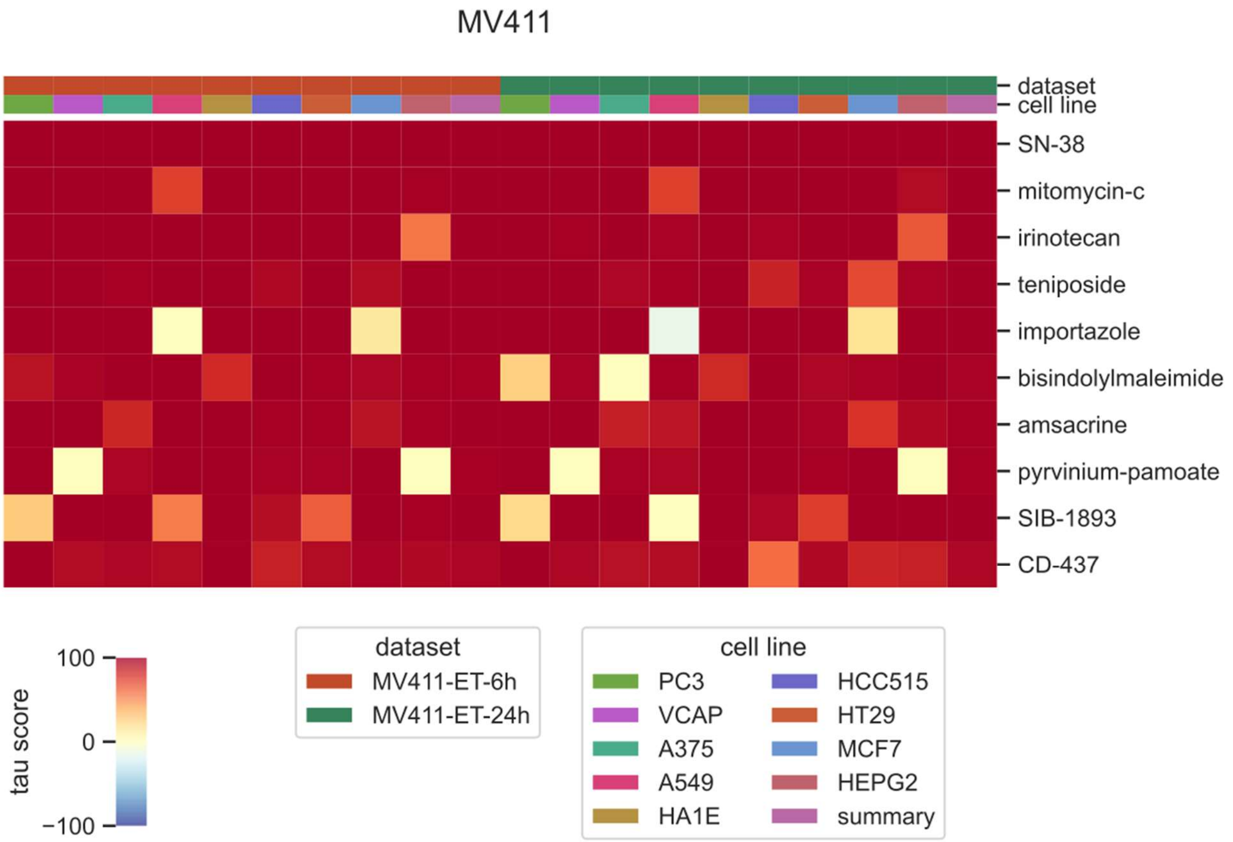
MV411
dataset
cell line
SN-38
mitomycin-c
irinotecan
teniposide
importazole
bisindolylmaleimide
amsacrine
pyrvinium-pamoate
SIB-1893
CD-437
tau score
100
0
−100
dataset
MV411-ET-6h
MV411-ET-24h
cell line
PC3
VCAP
A375
A549
HA1E
HCC515
HT29
MCF7
HEPG2
summary

E

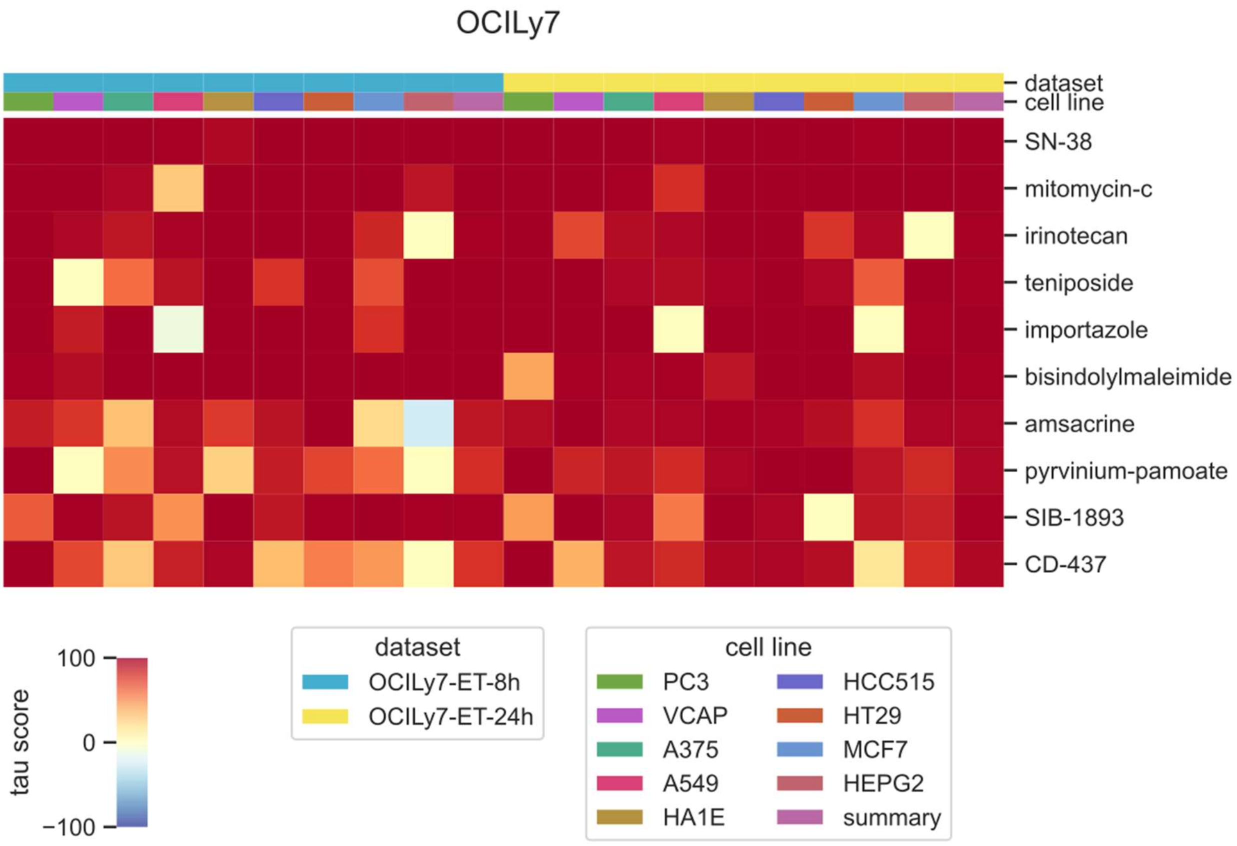
OCILy7
dataset
cell line
SN-38
mitomycin-c
irinotecan
teniposide
importazole
bisindolylmaleimide
amsacrine
pyrvinium-pamoate
SIB-1893
CD-437
tau score
100
0
−100
dataset
OCILy7-ET-8h
OCILy7-ET-24h
cell line
PC3
VCAP
A375
A549
HA1E
HCC515
HT29
MCF7
HEPG2
summary

F

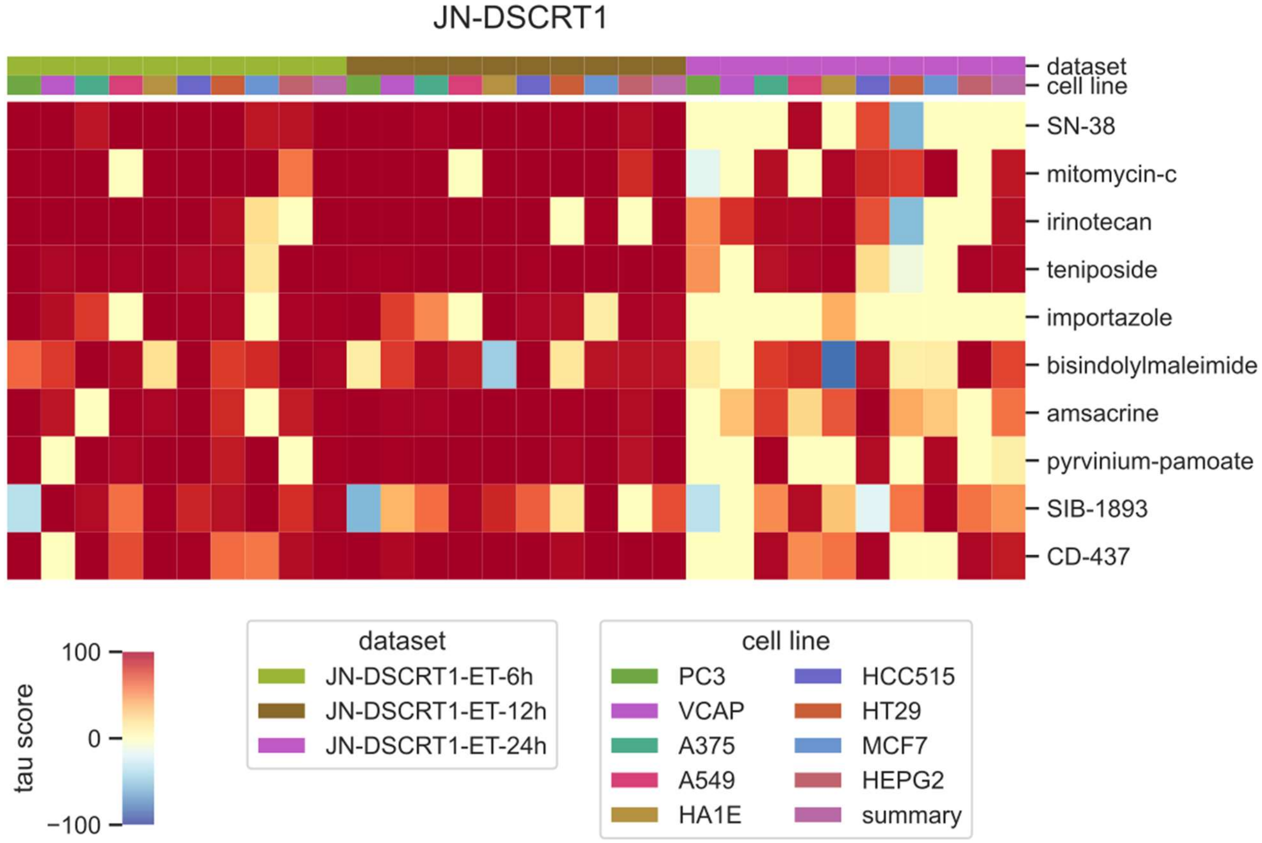
JN-DSCRT1
dataset
cell line
SN-38
mitomycin-c
irinotecan
teniposide
importazole
bisindolylmaleimide
amsacrine
pyrvinium-pamoate
SIB-1893
CD-437
tau score
100
0
−100
dataset
JN-DSCRT1-ET-6h
JN-DSCRT1-ET-12h
JN-DSCRT1-ET-24h
cell line
PC3
VCAP
A375
A549
HA1E
HCC515
HT29
MCF7
HEPG2
summary

G

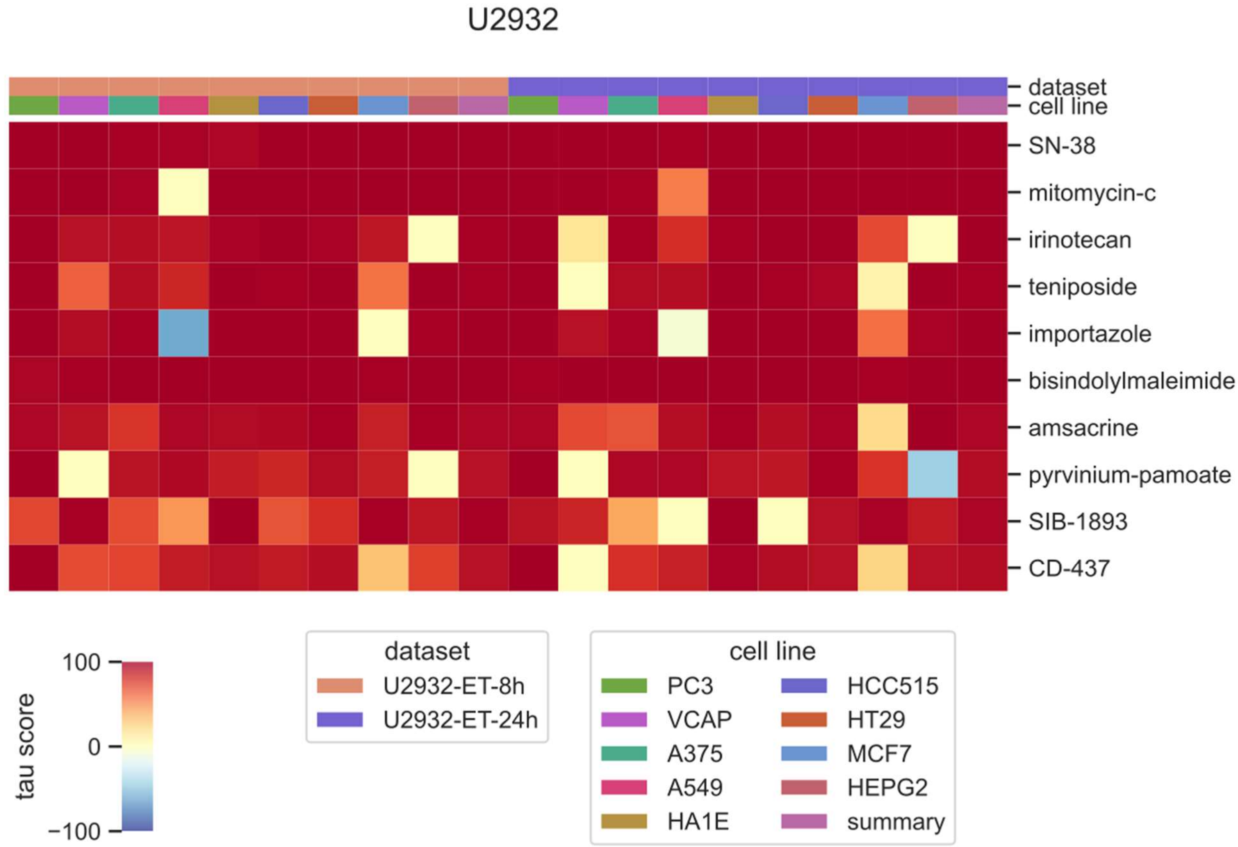
U2932
dataset
cell line
SN-38
mitomycin-c
irinotecan
teniposide
importazole
bisindolylmaleimide
amsacrine
pyrvinium-pamoate
SIB-1893
CD-437
tau score
100
0
−100
dataset
U2932-ET-8h
U2932-ET-24h
cell line
PC3
VCAP
A375
A549
HA1E
HCC515
HT29
MCF7
HEPG2
summary

H

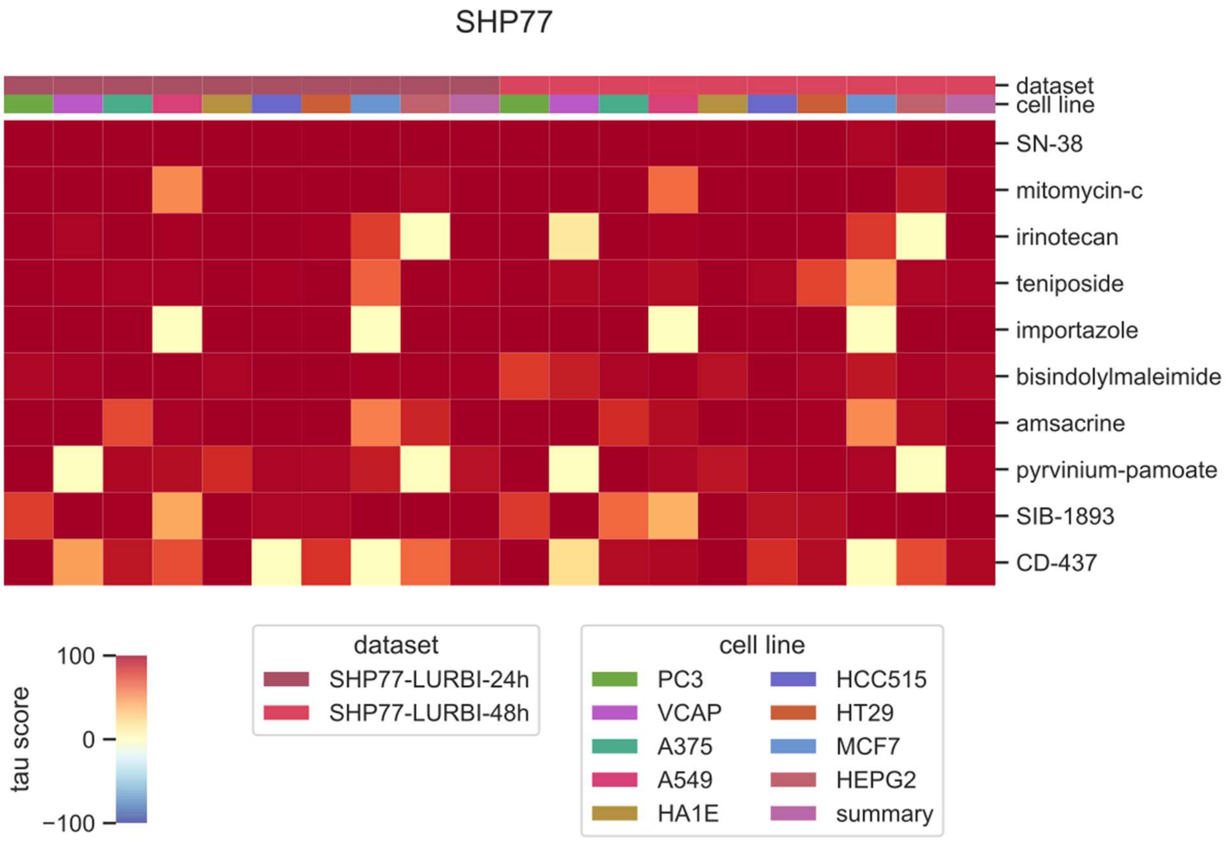
SHP77
dataset
cell line
SN-38
mitomycin-c
irinotecan
teniposide
importazole
bisindolylmaleimide
amsacrine
pyrvinium-pamoate
SIB-1893
CD-437
tau score
100
0
−100
dataset
SHP77-LURBI-24h
SHP77-LURBI-48h
cell line
PC3
VCAP
A375
A549
HA1E
HCC515
HT29
MCF7
HEPG2
summary
