# Supplementary material for: In-Silico Identification of Novel Pharmacological Synergisms: The Trabectedin Case
Source: Int J Mol Sci. 2024 Feb 8;25(4):2059. doi: 10.3390/ijms25042059 (PMC10888651; doi:10.3390/ijms25042059)

**Figure S2**

Panel A shows the clustering of the query signatures based on the common genes found through the comparison with gene profiles of the most connected compounds. Panel B shows the correlation between each pair of signatures. The correlation value is represented by colors as in the legend. Query signatures are sorted based on the clustering in panel A on the left.

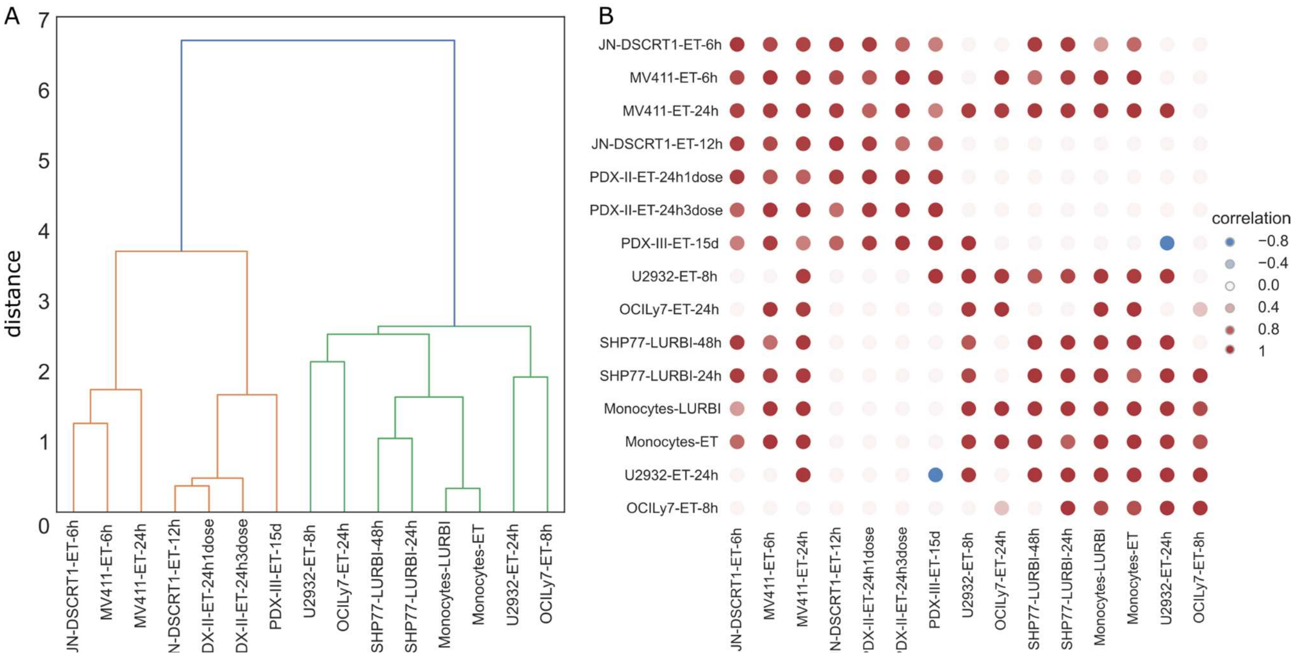

Supplement: Supplementary file 1 [file ijms-25-02059-s001.zip › Figure_S2.pdf]
